# Reaching Outside the Comfort Zone: Realising the FCTC’s Potential for Public Health Governance and Regulation in the European Union: Comment on "The Legal Strength of International Health Instruments – What It Brings to Global Health Governance?"

**DOI:** 10.15171/ijhpm.2017.107

**Published:** 2017-09-13

**Authors:** Florence Berteletti

**Affiliations:** Smoke Free Partnership, Brussels, Belgium.

**Keywords:** European Union (EU), Multilateralism, Tobacco Taxation, Public Health Governance

## Abstract

In their paper, Nikogosian and Kickbusch show how the effects of the adoption by the World Health Organization (WHO) of the Framework Convention on Tobacco Control (WHO FCTC) and its first Protocol extend beyond tobacco control and contribute to public health governance more broadly, by revealing new processes, institutions and instruments. While there are certainly good reasons to be optimistic about the impact of these instruments in the public health sphere, the experience of the FCTC’s implementation in the context of the European Union (EU) shows that further efforts are still necessary for its full potential to be realised. Indeed, one of the main hurdles to the FCTC’s success so far has been the difficulty in developing and maintaining comprehensive multisectoral measures and involving sectors beyond the sphere of public health.

The adoption by the World Health Organization (WHO) of the world’s first major international health treaty – the Framework Convention on Tobacco Control (FCTC)^1^– and later that of its first Protocol (the Protocol to Eliminate Illicit Trade in Tobacco Products),^2^ was a major political and legal victory in the fight against the tobacco epidemic. Indeed, the FCTC marks the agreement of its 180 parties on the principles and objectives concerning the approach to take on tobacco control, and moreover sets them down in a binding legal form. In so doing, it provides a solid foundation for all Parties to build upon in order to protect citizens from the devastating health, social, environmental and economic consequences of tobacco consumption and exposure to tobacco smoke,as well as a body in which states are obligated to report their progress to, based on the FCTCs recommendations.

However, in their perspective titled *“The Legal Strength of International Health Instruments – What It Brings to Global Health Governance?”* Nikogosian and Kickbush convincingly argue that the impact of the adoption of the FCTC and its first Protocol extends even beyond the domain of tobacco control, and has consequences for public health regulation in general.^3^ Indeed, the authors contend that these instruments have “*opened a new phase in WHO-global health that accepted internationally binding treaties as one major way forward and that they constituted a breakthrough by revealing new types of processes, institutions and instruments.*”

Although, as a public health expert and Director of the Smoke Free Partnership, I have regularly participated in the Conference of Parties and therefore have an international experience of FCTC negotiations, the major focus of my professional activity is primarily on its implementation at the level of the European Union (EU). This commentary will therefore show how some of the authors’ observations find confirmation to a great extent from the perspective of the implementation of the FCTC in the EU, while at the same time highlighting a central difficulty in ensuring the full efficacy of international health instruments such as the FCTC. Indeed, the benefits in terms of good public health governance and legislation postulated by the authors are often hampered by difficulties arising from a lack of multisectoral understanding of and action on the issues underpinning the FCTC.

It is worth noting that, as the EU is the only supranational body capable of negotiating and being a party to the FCTC on behalf of its Member States, it provides a unique forum in which to examine the question of the FCTC’s impact, as it comes with its own very specific set of processes, institutions and instruments.

## Progress Made and Impact at European Union Level

Prior to the ratification of the FCTC by the EU, the region was already among the forerunners of international tobacco control. Indeed, it first began regulating tobacco products as early as 1990 when it established minimum tar yields for cigarettes. Shortly after, this was complemented by the withdrawal of snus (tobacco for oral use) from the EU single market in 1992 following health concerns. However, the EU had been active in the field of tobacco control prior to these steps and during the 1980s and 1990s had introduced legislation governing smoke free workplaces, tobacco taxation, and tobacco advertising. In 2001, the EU adopted the first Tobacco Products Directive (TPD), which brought most of the existing tobacco product regulation under one legislative umbrella. The 2001 TPD strongly reflected the best available evidence at the time and marked a significant step forward for a number of Member States in the area of tobacco control. However, as scientific evidence continued to grow in the 2000s, it became clear that the 2001 TPD was becoming outdated.

The revision process of the TPD provides a prime example of how the FCTC has been used as a foundational springboard for further, more stringent laws and practices at EU level. Thus, the accession of the EU to the FCTC, and especially the visit of the former Head of the Convention Secretariat to the European Commission in 2012, was instrumental in obtaining a successful outcome. Indeed, this served as an opportunity to remind several high-level officials from different departments within the Commission of the EU’s obligations under the FCTC and, consequently, the revision process produced an exemplary piece of legislation which aligns very closely with the Convention.

Moreover, as noted in the commented article, examples of the policy-bolstering effects of the FCTC can also be observed at the national level, with several EU countries taking the initiative to go beyond the requirements of the Directive and adopt even stricter tobacco control laws, such as the plain packaging legislation introduced in Ireland, the United Kingdom, France, Hungary, and Slovenia.

Nevertheless, while the FCTC has been key to some crucial advances in tobacco control in the EU, significant efforts must still be made in terms of multisectoralism for the full benefits in terms of public health governance and regulation to take hold.

## Challenges Ahead

The WHO^4^ and the World Bank^5^ have identified the FCTC’s most effective measures which are supported by substantial evidence confirming their impact on reducing smoking prevalence.^6-9^ Furthermore, Article 4 of the FCTC calls on Parties to make a political commitment to develop and maintain comprehensive multisectoral measures (outside of the health sector), and to ensure the participation of civil society. Moreover, a global impact assessment report showed that Parties that have implemented multisectoral FCTC policies have generally experienced greater reductions in smoking prevalence.^10^ Indeed, in order to achieve a complete and effective tobacco control policy, the participation and collaboration of multiple sectors is necessary, as no single sector or agency can adequately address all elements of the FCTC. Thus, support of non-health sectors such as tax, customs, development, research, agriculture and trade is crucial, and the same is likely to be true for other public health policies. However, there is still some way to go in the EU for effective multisectoralism to be achieved. Whilst the EU is considered the global lead in tobacco tax policies, the implementation of Article 6 remains uneven and insufficient. A significant obstacle to the implementation of FCTC Article 6 (tax) in the EU has been the difference in levels of taxation between European countries as well as differences in levels of taxation between tobacco product categories. Another significant obstacle is institutional: whilst national Health Ministries might understand the positive benefits of higher tobacco prices on consumption levels, excise duties for tobacco products are determined by Ministries of Finance. Unfortunately, there is often little or no communication between the Health and Finance Ministries, resulting in the latter having little understanding of how important tobacco taxation can be in curbing smoking levels, and little appreciation of the full social and economic costs of smoking. The last obstacle is the interference of the tobacco industry in taxation policy: the tobacco industry has a strong interest in convincing governments to pursue lower-tax policies and it therefore invests considerable lobbying energy in weakening or defeating tobacco tax proposals; a key argument is that tobacco tax increases will lead to rises in illicit trade in tobacco products, and a decline in tax revenues. The tobacco industry supports its false economic arguments by commissioning reports which are rarely peer reviewed, generally of a lower quality when compared to reports written by independent academics, and often biased.^11-14^

Some Parties in the European region, such as the United Kingdom, France or the Ukraine^15^ have implemented Article 6 (for consistency) successfully. For example, in the United Kingdom, in 1993, Kenneth Clarke MP was the first Chancellor to explicitly state that he intended to raise the tax on tobacco for health reasons, noting that it was “*the most effective way to reduce smoking.”* Since then, apart from the period 2001-2008, successive UK governments have increased tobacco duties above the rate of inflation and the current commitment in place is for an escalation of 2% above inflation until 2020. Other successful examples include France, which began to substantially increase its tobacco tax in 1990, resulting in a three-fold increase in the inflation-adjusted price of cigarettes, and a reduction in cigarette consumption per adult per day of 50% (from about 6 to 3). Ukraine has also increased its excise taxes six times in 2010–2011 and increased revenues five times, along with a 26% decline in tobacco sales.

At EU level, the European Commission (EC) is currently considering the revision of the Tobacco Tax Directive (TTD) which defines the product categories, structure and minimum rates for excise duties on manufactured tobacco. In order to assess if the TTD is still fit for purpose, the Commission carried out a series of reports and evaluations to review the entire stock of EU legislation – to identify burdens, inconsistencies, gaps or ineffective measures and to make the necessary proposals to follow up on the findings of the review; In this context, Article 6 remains the FCTC’s least well-implemented aspect,^16^ despite the adoption of the FCTC Article 6 Guidelines, which were unanimously agreed at the 6th Conference of the Parties in 2014. This could be considered surprising, as measures recommended under Article 6 have been consistently shown by hundreds of studies from countries around the world to be one of the most effective for tobacco control. These studies confirm that increasing excise taxes on tobacco, which leads to higher tobacco prices, is by far the most effective tobacco control instrument to prevent cancer and chronic diseases, as well as the uptake of smoking by young people, and to re-balance health inequalities as part of a comprehensive tobacco control strategy^17-19^; all the evidence shows that implementation of Article 6 is very effective at combatting tobacco, and, as we have seen above, the Parties that have done it have been very successful on that level; it is all the more surprising given that the EU and the 28 Member States have all ratified the FCTC and have committed (and are legally bound) to its implementation, and are under the obligation – both under primary^20^ and secondary^21^ EU law – to ensure that fiscal legislation on tobacco products such as the TTD achieves a high level of health protection. In the case of the TTD revision, whilst it is certainly true that the FCTC did, to some extent, improve communication and cooperation between several of the Commission departments concerned – such as DG TAXUD (tax) and DG SANTE (health) this has not yet been translated into the various documents that have been published so far. How then can the apparent lack of impact of the FCTC on the TTD revision, compared to that of the TPD, be explained? Part of the answer can perhaps be found in the fact that the representatives negotiating the text in the Council of Ministers on behalf of the Member States are not health professionals, but tax attachés with little or no understanding of its health implications and the FCTC, who tend to perceive tobacco taxation as a money making method rather than a public health priority. Thus, although one of the aims of the directive is to align with the objectives of the FCTC, this results on a weaker focus on public health. This example illustrates how, even at the level of the EC and EU Member States, the FCTC is still struggling to be fully understood by decision-makers outside of health departments and public health non-governmental organizations (NGOs).

## Conclusion

If the principles and good practices contained in the FCTC are not reaching tax authorities and decision-makers outside of health departments, it will be difficult for international public health instruments do be developed and, perhaps more importantly, implemented successfully. A paradigm shift is definitely necessary for the dynamic that the FCTC has created to reach beyond the realm of tobacco control in a strict sense, as awareness on these issues has to be created from the bottom up. For this to happen, it is necessary for health departments in both the Member States and at EU level, as well as NGOs in the public health sector, to leave their comfort zone in order develop the necessary tools and contacts to communicate effectively with their counterparts in other areas of governance and create ownership of the principles contained in the FCTC within them. Only then will the conditions for new processes, institutions and instruments become fully possible and operational.

## Ethical issues

Not applicable.

## Competing interests

Author declares that she has no competing interests.

## Author’s contribution

FB is the single author of the paper.
